# Effect of Virtual Reality on Pediatric Pain and Fear During Procedures Involving Needles: Systematic Review and Meta-analysis

**DOI:** 10.2196/35008

**Published:** 2022-08-09

**Authors:** Marta Lluesma-Vidal, Raquel Carcelén González, Laura García-Garcés, María I Sánchez-López, Loreto Peyro, Cayetana Ruiz-Zaldibar

**Affiliations:** 1 Department of Nursing Faculty of Health of Science Universidad Cardenal Herrera-CEU, CEU Universities Alfara del Patriarca, Valencia Spain; 2 Department of Medicine and Surgery Faculty of Health of Science Universidad Cardenal Herrera-CEU, CEU Universities Alfara del Patriarca, Valencia Spain; 3 Department of Nursing Faculty of Health University of Camilo José Cela Villanueva de la Cañada, Madrid Spain

**Keywords:** virtual reality, pain, fear, pediatric, needle, child, injection, VR, systematic review, meta-analysis, paediatric

## Abstract

**Background:**

Virtual reality (VR) is used as a distraction measure during painful clinical procedures associated with the use of needles. These procedures include vaccinations, blood draws, or the administration of medications, which can cause children to feel increased levels of pain and fear.

**Objective:**

The objective of this study was to collect and analyze the current evidence regarding the effectiveness of VR as a tool to distract children from pain and fear during needle procedures as compared to that of standard techniques.

**Methods:**

A systematic review and meta-analysis was performed. We included randomized clinical trials (RCTs) or quasi-RCTs with participants younger than 21 years who underwent needle procedures in which the main distraction measure used was VR and where the main outcome measure was pain. The databases searched included the PubMed, Web of Science, Scopus, PsycINFO, CINAHL, and Cochrane libraries. In this systematic review, the studies were analyzed by applying the Critical Appraisal Skills Program guide in Spanish and the Jadad scale. In the meta-analysis, the effect size of the studies was analyzed based on the results for pain and fear in children.

**Results:**

From 665 unique search results, 21 studies were included in this systematic review, most of which reported low methodological quality. The study sample cohorts ranged from a minimum of 15 participants to a maximum of 220 participants. Ten studies were included in the meta-analysis. The global effect of using VR as a distraction measure was a significant reduction in pain (inverse variance [IV] –2.37, 95% CI –3.20 to –1.54; *Z*=5.58; *P*<.001) and fear (IV –1.26, 95% CI –1.89 to –0.63; *Z*=3.92; *P*<.001) in children in the experimental groups.

**Conclusions:**

The quality of the studies was mostly low. The main limitations were the impossibility of blinding the participants and health care personnel to the VR intervention. Nonetheless, the use of VR as a distraction measure was effective in reducing pain and fear in children during procedures involving needles.

## Introduction

### Background

The main problems experienced in pediatric care are pain and fear. This is especially true for procedures associated with the use of needles such as vaccinations, blood draws, or the administration of medications [1,2]. This causes difficulties in the administration of health care and can result in parental dissatisfaction [3]. The International Association for the Study of Pain defines pain as “an unpleasant sensory and emotional experience associated with actual or potential tissue damage or described in terms of such damage” [4]. Pain, therefore, is a complex experience that involves sensory, cognitive, behavioral, and psychological factors [5]. In turn, fear is an immediate alarm reaction to danger, which triggers an escape behavior and an intense physiological response [6]. The pain and fear that children experience when facing needle procedures is a concern for health care professionals. Therefore, various techniques are being studied to help reduce its impact. Indeed, the administration of drugs is not always indicated to reduce pain and fear in these procedures [7]. Rather, the use of distractions during painful procedures appears to be one of the most effective ways to decrease pain and distress in children [8]. For example, music or toys have already been effectively used as distraction measures to help reduce pediatric pain. Nonetheless, virtual reality (VR) is a novel technique that has been proven to be more effective than traditional methods [3].

VR is a computer technology that creates a 3D-simulated artificial environment [5]. It usually requires wearing special glasses that cover a wide field of vision and which include motion tracking systems at the eye level [9]. These glasses can be connected to a computer or a telephone [5]. VR makes it easier to divert attention away from the painful procedure so that children will have a slower response to pain signals by counteracting them with an experience of pleasant stimuli [10,11]. Several studies have evaluated the use of VR as a distraction measure during painful procedures such as venipuncture [3,12-15], tooth extraction [16-19], or burns treatment [20-24]. However, these studies have certain limitations such as the use of small sample sizes or poor methodological quality. Comparing the findings of these studies is difficult because the works published to date have evaluated a wide breadth of invasive medical care types. Furthermore, we were able to identify only 2 systematic reviews and 1 meta-analysis that analyzed the use of VR in children. However, these studies had evaluated several medical procedures, including dental procedures, burns treatments, oncological care, or physical therapy sessions [3,25]. The variation in the procedural conditions using VR implies a lack of evidence to support its use in needle procedures. Thus, highlighting these issues, this systematic review and meta-analysis focused on the effect of VR on pain and fear during needle procedures in children.

### Objectives

The general objective of this study was to collect and analyze the current evidence available regarding the effectiveness of VR as a tool to distract pediatric patients from potential pain and fear while undergoing needle procedures compared to the distractions by standard techniques. Regarding the specific objectives, our first aim was to analyze the studies included in the systematic review to assess their methodological quality. Second, our objective was to analyze the effect of the randomized controlled trials (RCTs) included in our meta-analysis.

### Research Question

Is the use of VR as a distraction measure effective for reducing the perception of pain in children while performing needle procedures?

## Methods

### Study Design

This is a systematic review and meta-analysis of studies that evaluated the effect of VR as the main distraction measure to reduce the perception of pain in children undergoing needle procedures.

### Inclusion Criteria

Studies were included in this paper based on the following criteria: (1) the participants were younger than 21 years; (2) studies where the use of VR was the primary distraction means used during needle procedures; (3) studies, including pilot studies, with an RCT or quasi-RCT methodological design; and (4) studies where the main outcome measure was pain.

### Data Sources

For this study, we consulted the PubMed, Web of Science, Scopus, PsycINFO, CINAHL, and Cochrane databases. The literature search was conducted between January 2020 and June 2021. Two independent researchers comprehensively reviewed the results obtained in each of the studies and subsequently compared the selected papers.

### Research Strategy

The medical subject heading keyword terms used in the search were reality, virtual, virtual reality, virtual reality headset, virtual reality exposure therapy, child*, pediatric, adolescent, intervention, program*, pain, ache, procedural, acute pain, pain perception, fear, and fears. All these terms were combined with the Boolean AND and OR functions and no filters were applied to limit the search. Search strategies were created specifically for each database by using the medical subject heading terms described above (Multimedia Appendix 1). No publication date or language restrictions were applied.

### Study Selection Process

First, we evaluated the scientific literature to identify studies that met the inclusion criteria. To do this, we read the title and abstract from each of the identified papers. Two of our authors (RCG and MLV) independently performed an initial screening by reading the study titles and abstracts. After this process, the researchers discussed their results based on the predetermined inclusion and exclusion criteria. There was a 6% discrepancy in the opinions of these authors, which was resolved by further discussion to reach a consensus.

### Data Extraction

Once the full-text papers were selected, 2 authors (RCG and CRZ) analyzed the studies based on their general characteristics and methodological quality. In this process, these researchers jointly extracted the relevant information from these publications. This information was transferred to 2 tables. First, the general characteristics of the studies were included in Multimedia Appendix 2. Subsequently, the methodological quality of all the studies was analyzed based on the Critical Appraisal Skills Program guide in Spanish (CASPe) scale, and this information was completed by performing a quantitative evaluation using the Jadad scale; these data are shown in Multimedia Appendix 3.

### Protocol and Registration

This systematic review was registered with the Open Science Framework (Osf.io/cd8nr) in October 2021.

### Data List

The general characteristics (Multimedia Appendix 2) of the studies provide information, including the following elements: author, study year and country, overall sample size, number of participants in the control and intervention groups, participant age, study type, variables and measurement instruments used, and finally, positive (*P*<.05), negative (*P*>.05), or inconclusive (±) results. Multimedia Appendix 3 provides an assessment of the methodological quality of the studies that we included in this review according to the CASPe [26]. This tool organizes data about each study into 3 sections: validity, results, and applicability. We used the Jadad scale [27], which assesses research quality on a scale of 0 to 5 points according to the responses to a series of questions, to complete this information. Scores below 3 points suggested that little methodological rigor had been applied during the study in question. This allowed us to objectively assess the following parameters: random sequence generation, allocation concealment, blinding of participants and personnel, and blinding to the outcome assessment. To guarantee the quality of this meta-analysis, we followed the PRISMA (Preferred Reporting Items for Systematic Reviews and Meta-Analysis) statement guidelines [28] (Tables S4 and S5 of Multimedia Appendices 4 and 5, respectively).

### Risk of Bias Assessment

The Cochrane Collaboration Risk of Bias Tool [29] was used to assess the risk of bias in the studies included in the meta-analysis in 5 categories: selection bias, performance bias, detection bias, attrition bias, and reporting bias. For selection bias, which refers to the introduction of differences between groups at baseline, random sequence generation and allocation concealment were judged. Performance bias was analyzed based on blinding of the participants and personnel. Detection bias referred to blinding of the outcome assessors. Attrition bias included different rates of withdrawals between groups and was judged according to the proportion of incomplete outcome data. Finally, reporting bias described selective reporting.

The Cochrane Collaboration Handbook for Systematic Reviews for Interventions was used to analyze the risk of bias from studies not included in the meta-analysis. This analysis included selection bias when randomization was analyzed, performance bias when blinding between participants and personnel was tested, detection bias when blinding between participants and outcome assessors was tested, attrition bias where dropouts were analyzed, and reporting bias where they were analyzed, and the outcomes were selectively reported [29].

### Analysis of the Meta-analysis Data

Employing the random effects model in Review Manager software (RevMan v.5.2; Cochrane Collaboration), 2 meta-analyses were carried out to examine the overall effect of the intervention on pain and fear in children. We used this model because we wanted to limit overestimation of the effect size. The studies included had an RCT design and contained complete statistical information; the effects were expressed as mean differences with a 95% CI. The heterogeneity of the studies was assessed by calculating the *I*^2^ statistic, and the variance between the studies was examined by calculating Tau^2^. When the significance level was set at .05, the heterogeneity of the studies we included was high for both these variables (94% and 96%, respectively; *P*<.01). Lastly, to increase the precision of the effect size estimator, the effect sizes proposed by Cohen [30] were calculated (small effect, *d*=0.20; medium effect, *d*=0.50; and large effect, *d*=0.80).

## Results

### Search Results

**Figure 1:**
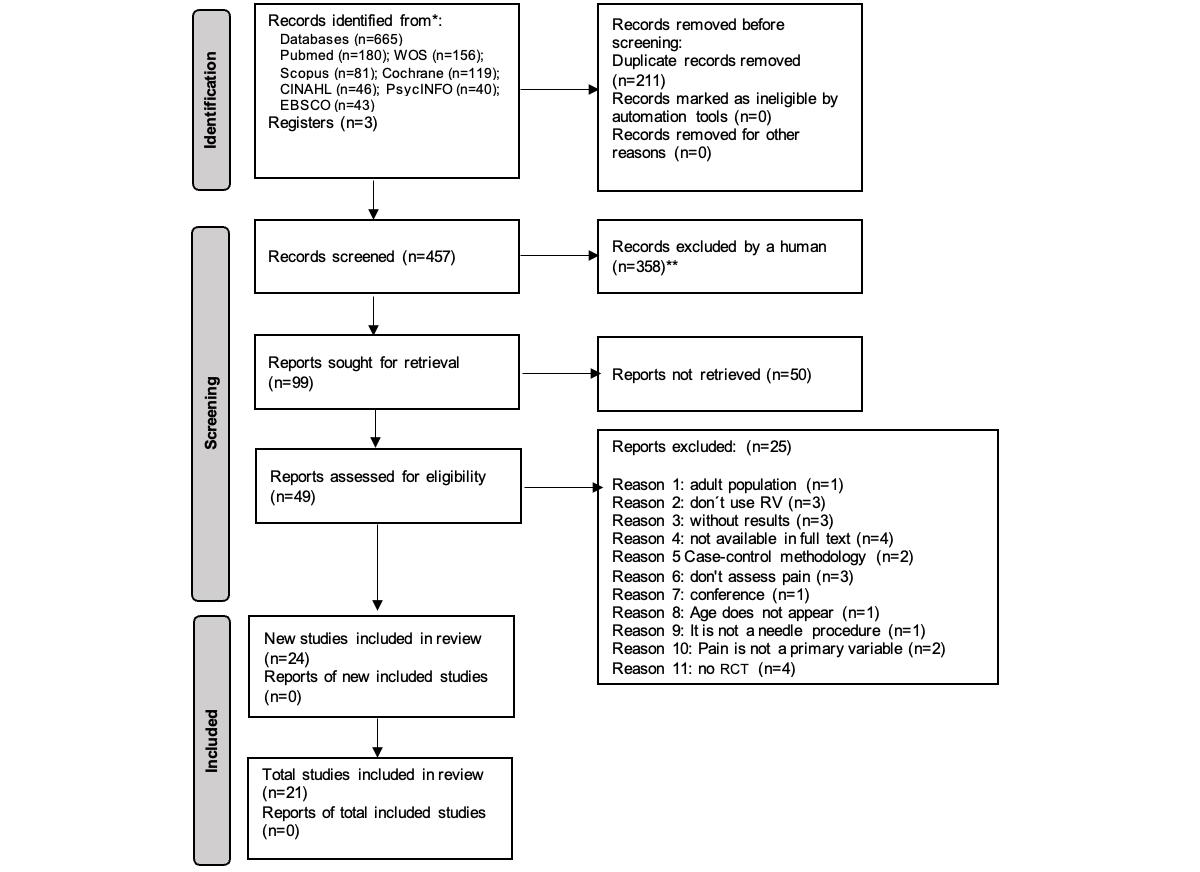
Flowchart showing the screening and selection process for the papers included in this systematic review and meta-analysis. Created using the guidelines on Page et al [31]. WOS: Web of Science; *Consider if feasible to do so, reporting the number of records identified from each database or register searched (rather than the total number across all databases/registers); **If automation tools were used, indicate how many records were excluded by a human and how many were excluded by automation tools.

As shown in Figure 1, our initial search returned a total of 665 papers. After eliminating 211 duplicates, 2 researchers (RCG and MLV) initially screened the 454 studies by reading their titles and abstracts. There was a 6% discrepancy in their opinions, which was resolved by reaching a consensus based on the eligibility criteria of the papers. This selection further reduced the sample to 96 manuscripts. Reading the full texts of these papers revealed that only 46 papers had focused on the use of VR to reduce pain during procedures involving needles, some of which had also addressed fear in these patients. Lastly, 3 of our authors (RCG, MLV, and CRZ) critically read all these papers and excluded another 25 papers because they did not meet the inclusion criteria, as described in Figure 1. Thus, 21 studies were finally included in this systematic review, and only 10 were eligible for inclusion in the meta-analysis [31] (Figure 1).

### General Characteristics of the Studies

Multimedia Appendix 2 summarizes the main characteristics and results of the selected studies. The 21 studies ranged from the year 2002 to 2021; 76% (16/21) of them had been published between 2018 and 2021 [32-47], while the rest had been published between 2002 and 2007 [48-52]. Most of the research (11/21, 52%) had been conducted in North America [33,35-37,39,40,48-52], but 14% (3/21) of the work was from Europe [34,35,37] and 33% (7/21) had been performed in Asia [32,36,38-40,44,47]. Regarding the contexts of these VR studies, 95% (20/21) of them had been carried out in hospitals [32-50,52], while 5% (1/21) had been completed in primary health care centers [51]. Most of the procedures involving needles in which VR had been used were venipunctures (14/21, 67%) [32,34,35,37-43,45,47,49,51], followed by subcutaneous venous puncture for reservoir-type venous access (4/21, 19%) [33,36,48,52]. One study had used VR during lumbar punctures [50]. The remaining one had conducted research analyzing venipunctures or intramuscular injections [42]. The study sample cohorts ranged from a minimum of 15 [41] to a maximum of 220 individuals [33]; 43% (9/21) of the studies had analyzed a sample comprising fewer than 100 participants [35,37,41-43,48,49,51,52]. In the different studies, the age of the children ranged from 4 years to 21 years, while the study duration varied between 14 weeks and 20 months; 57% (12/21) of the studies collected data for less than a year [32,34-37,39-41,43,44,46,47], 10% (2/21) had done so for 13 months or more [33,45], and 33% (7/21) of them had not reported this information [38,42,48-52]. We identified most of the studies (16/21, 76%) as RCTs [32-34,36-40,42-47,49,52] but 10% (2/21) were pilot studies [48,50] and 10% (2/21) were quasi-experimental studies [35,41].

### Quality of the Studies

We assessed the quality of the studies according to the CASPe and Jadad guidelines (see Multimedia Appendix 3). Only 14% (3/21) of the studies were rated as high quality [37,44,45], with the remaining 86% (18/21) being rated as low quality [32-36,38-43,46-52]. Specifically regarding the random assignment of patients to the study groups, 10% (2/21) [34,35] of the studies had not carried out randomization. The participants had been randomly assigned in 19% (4/21) of the studies [32,49,50,52] but none of these authors had specified the type of procedure they had used to perform the randomization, and this information was uncertain in another study (1/21, 5%) [41]. The participants had been randomly assigned in the remaining 71% (15/21) of the studies [33,36-40,42-48,51]. Given the active nature of these interventions, most of the studies had not blinded the participants to their group assignment. Moreover, only 4 (19%) of them [37,39,45,52] had blinded the group assignment to the observers or health professionals, although none of them had been able to maintain this blinding until the end of the work. The preintervention VR and control group characteristics were similar in terms of sex, age, and other sociodemographic variables in 15 (71%) of the 21 studies [32,33,36-39,42-45,47-49,51,52]. In 91% (19/21) of the cases, both groups had been treated in a similar way, regardless of the intervention that had been performed [32,33,35-39,41-45,47-53]. There were insufficient reports on the flow of participants through the studies in 38% (8/21) of the papers retrieved, which made it difficult to determine the level of dropouts [34,41,42,48-52]. Only 1 study (5%) provided information about the effect size [33]. The cohorts comprised 15 to 59 children in 43% (9/21) of the studies, and the authors themselves classified these samples as small [35,41-43,48-52]. Furthermore, 5% (1/21) of the samples were of children with specific pathologies [34]. Regarding extrapolation of the results, the data could only be generalized or considered for extrapolation in 8 of the 21 papers we reviewed [32,33,37,39,40,43-45]. Additionally, only 43% (9/21) of the studies had collected information about the participant acceptance and satisfaction with the VR intervention [32,35,39-41,44,46-48]. Finally, the benefits of the intervention had exceeded the costs or damages that could have been produced in all of the cases [32-52].

### Risk of Bias

**Figure 2:**
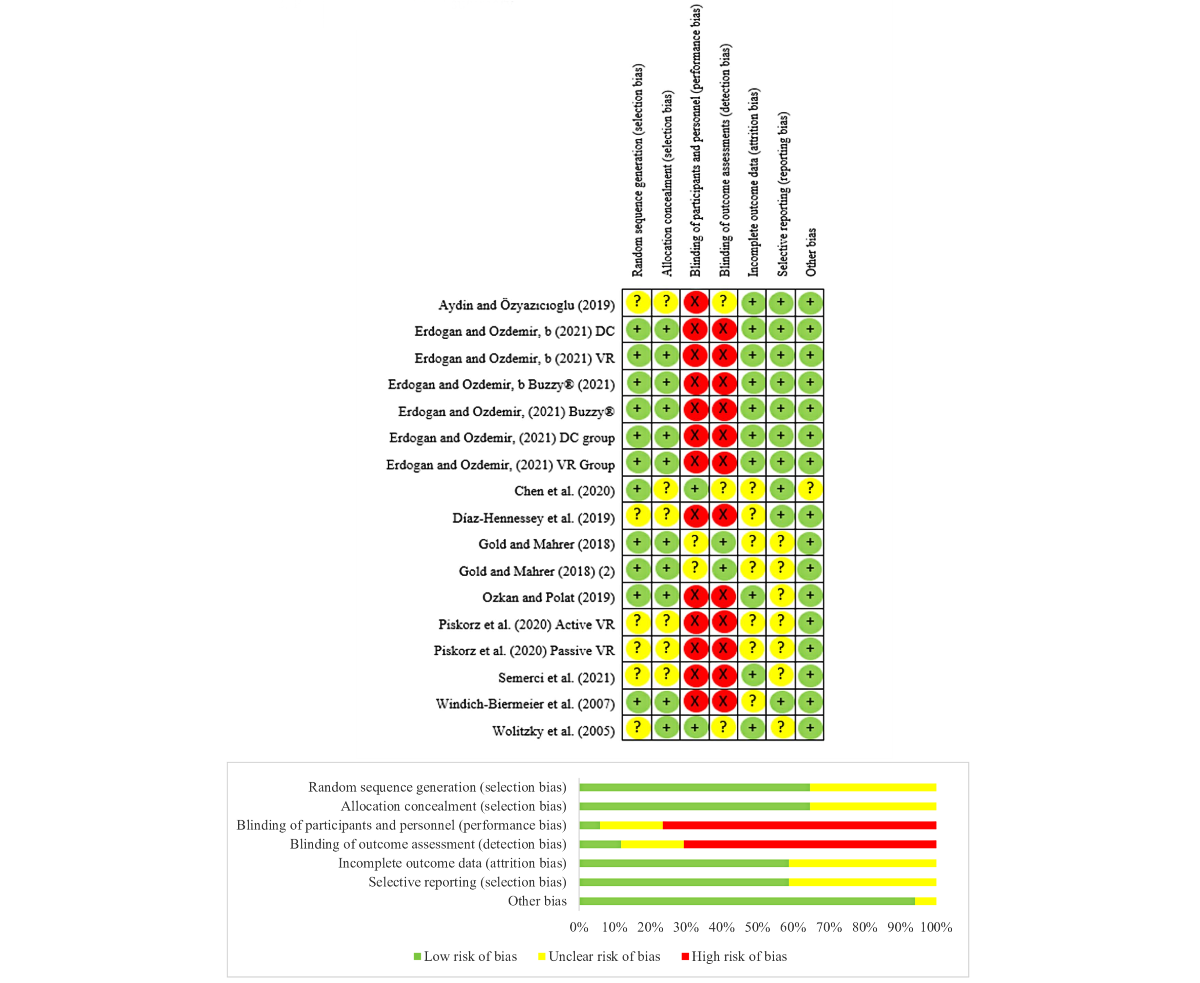
Risk of bias graph and summary [34,36,40,41,43,45,47,51,52].

The Cochrane Collaboration Risk of Bias Tool [29] was used to assess the risk of bias of the 10 studies included in the metanalysis by 2 reviewers. Based on these tools, only 1 of the studies was at high risk of bias, 8 at unclear risk of bias, and 1 at low risk of bias (Figure 2). Based on the Cochrane Collaboration criteria for different types of bias, we analyzed the 11 studies not included in the meta-analysis. As shown in Multimedia Appendix 3, the biases related to blinding, both of the participants of the personnel as well as to the outcome assessment, reached the highest levels in 82% (9/11) of the studies. Of the 11 studies, most of the studies had a moderate risk of bias (5/11, 46%); 3 (27%) studies were identified as having a high risk of bias and 2 (18%) studies had a low risk of bias. One study (9%) was classified as having a low risk of bias but no information on blinding could be obtained.

### Effects of VR on the Perception of Pain

**Figure 3:**
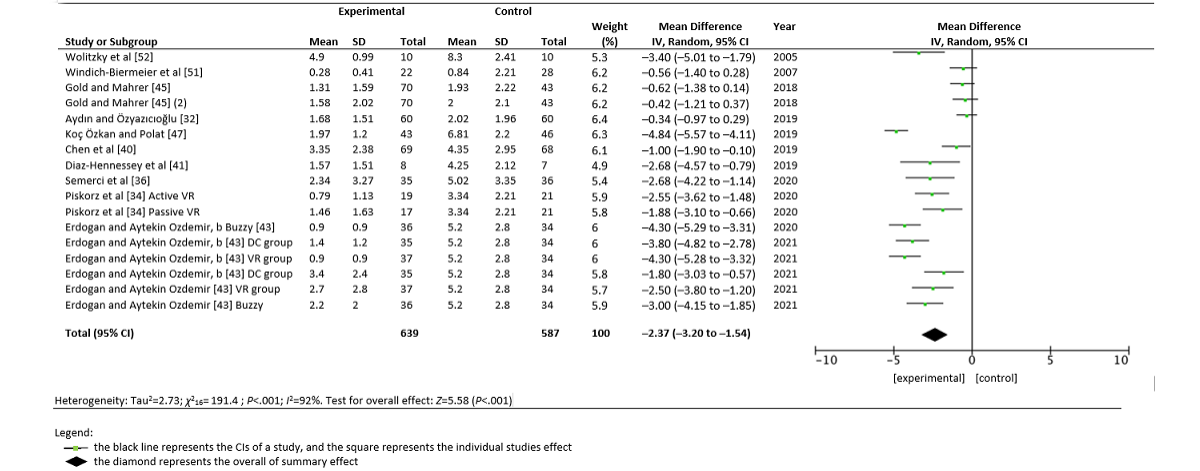
A random forest plot of the association between pain and study group (control vs virtual reality) [34,36,40,41,43,45,47,51,52]. b: Wong-Baker Faces Pain Rating Scale; Buzzy: a device that applies local cold and vibration at the injection site; DC: distraction card; IV: inverse variance; VR: virtual reality.

The studies were heterogeneous in both the measured outcomes (*I*^2^=89-92). We were able to analyze the effect size of the pain studies in 10 of the 21 studies (Figure 3). The main results showed statistically significant differences in favor of the experimental group in the studies by Wolitzky et al [52] (*d*=1.85; inverse variance [IV] –3.40, 95% CI –5.01 to –1.79) and Diaz-Hennessey et al [41] (*d*=1.43; IV –2.68, 95% CI –4.57 to –0.79). Likewise, pain was significantly reduced in the studies by Koç Özkan and Polat [47] (*d*=0.17; IV –4.84, 95% CI –5.57 to –4.11), the intervention by Piskorz et al [34] using both passive VR (*d*=0.97; IV –1.88, 95% CI –3.10 to –0.66) and active VR (*d*=1.45; IV –2.55, 95% CI –3.62 to –1.48), and in the studies by Erdogan and Aytekin Ozdemir [43] in VR versus a control group (*d*=0.89; IV –2.5, 95% CI –3.80 to –1.20). The study by Chen et al [40] also found a significant reduction in pain in the intervention group (*d*=0.37; IV –1.00, 95% CI –1.90 to –0.10). As shown in Figure 3, the global effect of using VR as a distraction measure had significantly reduced pain in children in the experimental groups (IV –2.37, 95% CI –3.20 to –1.54; *Z*=5.58; *P*<.001).

### Effects of VR on Fear

**Figure 4:**
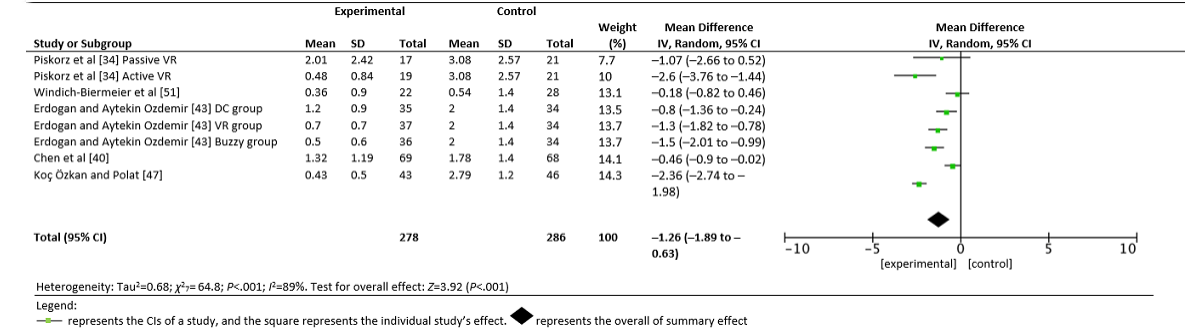
A random forest plot of the association between fear and study group (control vs virtual reality) [34,40,43,47,51]. Buzzy: a device that applies local cold and vibration at the injection site; DC: distraction card; IV: inverse variance; VR: virtual reality.

We were only able to analyze the fear variable in 5 of the 21 studies. The use of VR produced a statistically significant reduction in fear in the experimental groups in the study by Chen et al [40] (*d*=0.35; IV –0.46, 95% CI –0.90 to –0.02) and a large reduction in the Koç Özkan and Polat study [47] (*d*=0.17; IV –2.36, 95% CI –2.74 to –1.98). Likewise, fear was significantly reduced in the studies by Erdogan and Aytekin Ozdemir [43] in the VR versus control group (*d*=1.17; IV –1.30, 95% CI –1.82 to –0.78) and the intervention by Piskorz et al [34] in active VR (*d*=1.36; IV –2.60, 95% CI –3.76 to –1.44]. As shown in Figure 4, the global effect of using VR as a distraction measure had significantly reduced the perception of fear in children in the experimental groups (IV –1.26, 95% CI –1.89 to –0.63; *Z*=3.92; *P*<.001).

## Discussion

To the best of our knowledge, this is the first systematic review with a meta-analysis designed to examine the effectiveness of the use of VR as a distraction measure to reduce pain and fear in the pediatric population during procedures involving needles. Based on the high effect sizes that we found, our results suggest that VR distraction is possibly more effective than the habitual routine or other distractions used during needle procedures to reduce the perception of pain and fear felt by children. It is difficult to compare these results with those of other studies because most of them included different medical processes or did not analyze the effect on the children’s fear. However, other meta-analyses found similar results, indicating that the effects of VR are beneficial in reducing fear during medical processes involving pain, especially in children [54]. However, these comparisons must be analyzed with caution because neither the studies included nor their participants were homogeneous in terms of age or characteristics, the medical procedures analyzed, or the tools used to measure pain.

Most of the papers included in this review found that VR had a positive effect by helping to reduce pain in children. Of note, all the studies that had included more than 100 participants and had used the Wong-Baker Faces Pain Rating Scale (WBFPS) had reported statistically significant results. This may be because this visual assessment scale is more effective in assessing pain in children than other scales that use numerical assessment scales such as the visual analog scale (VAS) for pain [55]. Although the VAS is a reliable method for assessing acute pain, children younger than 7 years may have difficulty in its use, as indicated by the reduced reliability of the results reported in these studies [56]. In addition, the VAS and WBFPS have been widely used in studies evaluating pain in other procedures such as wound healing [57], physiotherapy sessions after complex surgical interventions [58], or dental procedures [59] in which they produced positive results.

Most of the papers included in this review [32,33,35,37-41,44,45,47-52] had analyzed the effect of VR on pain and fear in pediatric patients with cancer during venipuncture or reservoir puncture procedures. Furthermore, most of the studies we retrieved (20/21, 95%) had been carried out in hospitals, while only 5% (1/21) had been carried out in primary health care centers. This may have been a result of the health care provision resources available at the sites where these previous studies had been carried out, given that most of this work had been carried out in hospitals, thanks to the teaching function of these centers [60-62]. These data indicate that scant research has been carried out for this level of care, which is surprising, considering that needle procedures are frequent in primary care contexts because of the systematic vaccination programs carried out in the pediatric population. Among other possible explanations, perhaps this lack of research can be explained by health care staff overload or low levels of motivation among professionals or toward the support of research [63-67]. However, 2 study protocols have recently been published that will aim to evaluate the effectiveness of VR against pain during vaccination in the pediatric population through RCTs with estimated sample sizes of 100 [68] to more than 400 participants [69].

Although we found that VR is effective in reducing children’s fear, very few studies have demonstrated the usefulness of VR in reducing fear during procedures involving needles [40,47]. Thus, the absence of a validated scale to measure this variable may be inhibiting its proper evaluation [70]. According to Taddio et al [71], most studies that measure fear do so by using questionnaires developed by the investigators, nonvalidated scales, or scales for measuring anxiety [72,73]. Thus, this review reveals the lack of consensus on the most appropriate instruments for evaluating and clearly differentiating between fear and distress in the pediatric population. Although in clinical practice, the difference between fear, anxiety, and stress may not always be relevant, these represent different theoretical constructs, which are not always rigorously differentiated. Notwithstanding, both fear and distress are important factors that are related to and impact the pain perceived by children [74,75].

Of note, the quality of the studies included in this systematic review (based on CASPe and Jadad assessments) was mostly low. However, some studies with low quality or even small samples showed important effects. We assume that in the future, a meta-regression model could be used to expand existing knowledge about these intervention types and their methodological quality. For this reason, this systematic review and meta-analysis highlights the need to design and implement new research with high methodological quality that would allow extraneous variables to be isolated, favoring the cause-effect relationship. The principal reasons for the studies included in this meta-analysis to be of low quality were that it was nearly impossible to blind both the participants and health care personnel to the VR intervention because of the nature of these devices [76]. Furthermore, in many cases, the absence of randomization was justified for ethical reasons. Indeed, more than half of the studies we examined had considered small sample sizes of fewer than 100 participants [77], which, in addition to being unreliable and inefficient, can lead to overestimation of the study effect size and can produce low reproducibility of the results. Finally, chronological age and neurological development are among the factors that influenced children’s perceptions of pain and fear of procedures involving needles, and therefore, adjusting the age of children to less than 21 years should be considered in future studies [78]. Blinding and randomization are also the issues that were identified in the risk of bias analysis of studies not included in the meta-analysis. The studies included in the meta-analysis generally had a low level of risk, while studies not included tended to have a higher level of risk of bias. This may be due both to the fact that meta-analysis studies are more robust and to the use of different measurement tools in these papers.

The main limitations of this work were, on the one hand, the lack of studies with nonsignificant results available in the scientific literature. This meant that we may not have included all the relevant studies, and therefore, it was not possible to control for publication bias [79]. On the other hand, although the random effects model that we used favored the most realistic observation of the data by specifically weighting each study, the heterogeneity of the included studies, both in terms of their outcome measures and their methodological approaches, means that we must be cautious about the interpretation of our results. This problem was also identified in a similar recent meta-analysis in which heterogeneity was found in studies with young patients [54]. Finally, the studies included did not address the effect of VR in children younger than 4 years, which implies a limitation of the results when it comes to generalizing this effect in all children. Based on all the above, the methodological design of future work must adequately calculate the required sample sizes and use appropriate sampling, participant study group allocations, and blinding techniques to be able to extrapolate any data obtained to the wider pediatric population. This review was limited by the quality of the studies it included. Generalization of these findings to younger children should also be done with caution because the studies we considered had not included children younger than 4 years.

In conclusion, the findings of this review indicate that VR could be a feasible distraction measure to reduce the perception of pain and fear in the pediatric population during procedures involving needles. However, these results are limited by the heterogeneity of the studies included. In this sense, more trials with larger sample sizes and quality methodological techniques will be needed in the future.

## Appendix

Multimedia Appendix 1 Search strategies.

Multimedia Appendix 2 General characteristics and results of the studies in this review.

Multimedia Appendix 3 Evaluation of the methodological quality.

Multimedia Appendix 4 PRISMA (Preferred Reporting Items for Systematic Reviews and Meta-Analysis) checklist.

Multimedia Appendix 5 PRISMA (Preferred Reporting Items for Systematic Reviews and Meta-Analysis)_2020_Abstract_checklist.

## Abbreviations

CASPe: Critical Appraisal Skills Program guide in Spanish
IV: inverse variance
RCT: randomized controlled trial
VAS: visual analog scale
VR: virtual reality
WBFPS: Wong-Baker Faces Pain Rating Scale
