# Supplementary material for: Effect of Virtual Reality on Pediatric Pain and Fear During Procedures Involving Needles: Systematic Review and Meta-analysis
Source: JMIR Serious Games. 2022 Aug 9;10(3):e35008. doi: 10.2196/35008 (PMC9399850; doi:10.2196/35008)
Supplement: Multimedia Appendix 1 [file games_v10i3e35008_app1.doc]

**Multimedia Appendix 1. Search strategies**

| **Database** | **Search Strategy** |
| --- | --- |
| **PubMed** | ((("Pain, Procedural"[Mesh]) AND "Virtual Reality"[Mesh]) AND ("Child"[Mesh] OR "Child, Preschool"[Mesh])) AND "Adolescent"[Mesh]))) |
| **Web of Science**  **(WOS)** | [(Reality, Virtual OR virtual reality OR virtual reality headset OR virtual reality exposure therapy)] AND [(child* OR pediatric OR adolescent)] AND [(intervention OR program*)] AND [(pain OR ache OR pain procedural OR acute pain OR pain perception)] |
| **Scopus** | [(Reality, Virtual OR virtual reality OR virtual reality headset OR virtual reality exposure therapy)] AND [(child* OR pediatric OR adolescent)] AND [(intervention OR program*)] AND [(pain OR ache OR pain procedural OR acute pain OR pain perception)] |
| **PsycINFO** | [(Reality, Virtual OR virtual reality OR virtual reality headset OR virtual reality exposure therapy)] AND [(child* OR pediatric OR adolescent)] AND [(intervention OR program*)] AND [(pain OR ache OR pain procedural OR acute pain OR pain perception)] |
| **Cuiden** | [(Reality, Virtual OR virtual reality OR virtual reality headset OR virtual reality exposure therapy)] AND [(child* OR pediatric OR adolescent)] AND [(intervention OR program*)] AND [(pain OR ache OR pain procedural OR acute pain OR pain perception)] |
| **Ebsco** | [(Reality, Virtual OR virtual reality OR virtual reality headset OR virtual reality exposure therapy)] AND [(child* OR pediatric OR adolescent)] AND [(intervention OR program*)] AND [(pain OR ache OR pain procedural OR acute pain OR pain perception)] |
| **Cochrane** | pain procedural in Title OR acute pain in Title OR pain perception in Title AND virtual reality in Title OR virtual reality headset in Title OR children in Title OR pediatric in Title |
