# Supplementary material for: Effect of Virtual Reality on Pediatric Pain and Fear During Procedures Involving Needles: Systematic Review and Meta-analysis
Source: JMIR Serious Games. 2022 Aug 9;10(3):e35008. doi: 10.2196/35008 (PMC9399850; doi:10.2196/35008)
Supplement: Multimedia Appendix 2 [file games_v10i3e35008_app2.doc]

**Multimedia Appendix 2. General characteristics and results of the studies in this review.**

| **Author**  **Year**  **Country** | **Procedure and setting** | **Participants (*N*)**  **Age** | **Study length** | **Study design** | **VR equipment**  **Brand: model (software/program)** | **Tools**  **Variables** | **Results** |
| --- | --- | --- | --- | --- | --- | --- | --- |
| Wint et al, 2002 [50]  **USA** | Lumbar puncture.  Hospital. | *n* = 30 (IG^a^: 17; CG^b^: 13).  10–19 years. | N/A^c^ | RTC^d^. | i-O Display Systems LLC: Menlo Park, CA: VR^e^ glasses  (video entitled ‘Escape’, distributed by VIRTUAL i-O). | **Pain:**  VAS^f^. | – |
|  |  |  |  |  |  | **Sedation:**  Sedation Assessment Scale. | – |
|  |  |  |  |  |  | **Subjective evaluation of the virtual experience:**  Researcher developed questionnaire. | Qualitative results. |
| Gershon et al, 2004 [48]  **USA** | Subcutaneous reservoir puncture.  Hospital. | *n* = 59 (IG^a^: 22; Non-VR^d^: 22; CG^b^: 15).  7–19 years. | N/A^c^ | RTC^d^. Pilot study. | NAV^g^: NAV^g^ (Virtual Gorilla program, Georgia Institute of Technology’s SVE^v^). | **Pain:**  VAS^f^. | – |
|  |  |  |  |  |  | **Anxiety:**  VAS^f^.  CHEOPS^h^. | –  – |
|  |  |  |  |  |  | **Pulse Rate:**  Pulse oximeter. | ± |
| Wolitzky et al, 2005 [52]  **USA** | Subcutaneous reservoir puncture.  Hospital. | *n* = 20 (IG^a^: 10; CG^b^: 10).  7–14 years. | N/A^c^ | RTC^d^. | NAV^g^: NAV^g^ (Virtual Gorilla program, Georgia Institute of Technology’s SVE^v^). | **Anxiety:**  How I feel questionnaire.  VAS^f^. | –  + |
|  |  |  |  |  |  | **Pulse Rate:**  Pulse oximeter. | + |
|  |  |  |  |  |  | **Pain:**  VAS^f^.  CHEOPS^h^. | ±  + |
|  |  |  |  |  |  | **Thoughts and feelings.**  Interview. | Qualitative results. |
| Gold et al, 2006 [49]  **USA** | Venipuncture.  Hospital. | *n* = 20 (IG^a^: 10; CG^b^: 10).  8–12 years. | N/A^c^ | RTC^d^. | Logitech rumble pad: 5DT HMD 800.  (Street Luge, 5DT with Microsoft Windows XP). | **Pain:**  VAS^f^  WBFPS^i^.  FPS-R^j^. | –  +  – |
|  |  |  |  |  |  | **Anxiety:**  Childhood Anxiety Sensitivity Index. | – |
|  |  |  |  |  |  | **Perception of illness:**  Child Simulator Sickness Questionnaire. | – |
|  |  |  |  |  |  | **Commitment to the intervention:**  Child Presence Questionnaire. | – |
|  |  |  |  |  |  | **Satisfaction of parents, children, and nurses:**  Likert-format surveys. | + |
| Windich-Biermeier et al, 2007 [51]  **USA** | Subcutaneous reservoir puncture. Venipuncture.  Primary Healthcare Centres | *n* = 50 (IG^a^: 22; CG^b^: 28).  5–18 years. | N/A^c^ | Quasi-experimental study. | Distraction 1: Super challenger book.  Distraction 2: Bubbles; parents blew bubbles using a nontoxic solution.  Distraction 3: Disney music.  Distraction 4: i-O Display Systems LLC, VIRTUAL i.o. (Portland, OR).  Distraction 5: Nintendo® Gameboy Advance® | **Pain:**  CAS^k^. | – |
|  |  |  |  |  |  | **Fear:**  Glasses Fear Scale. | – |
|  |  |  |  |  |  | **Stress:**  OSBD^l^. | – |
|  |  |  |  |  |  | **Venipuncture experience:**  IPQ^m^. Children and parents. | + |
| Gerçeker et al, 2018 [39]  **Turkey** | Venipuncture.  Hospital. | *n* = 121 (IG^a^ – VR^e^: 40; IG^a^ -exter NAV^g^ l cold, and Vibration: 41; CG^b^: 40).  7 –12 years. | 5 months. | RTC^d^. | G1: Samsung Gear Oculus headset with a Samsung Galaxy S5 Note phone.    G2: Buzzy® (MMJ Labs, Atlanta, GA). | **Pain:**  WBFPS^i^. | + |
| Gold and Mahrer, 2018 [45]  **USA** | Venipuncture.  Hospital. | *n* = 143 (IG^a^: 70; CG^b^: 73).  10–21 years. | 13 months. | RTC^d^. | Different VR^e^ goggles were used.  NAV^g^ (VR^e^ game Bear Blast applied using a Samsung Galaxy S6). | **Pain and Anxiety:**  CAS^k^.  VAS^f^.  FAS^o^. | +  +  + |
|  |  |  |  |  |  | **Anxiety sensitivity:**  CASI^p^. | + |
|  |  |  |  |  |  | **Satisfaction:**  Researcher developed questionnaire. | N/A^c^ |
| Piskorz and Czub, 2018 [35]  **Poland** | Venipuncture.  Hospital. | *n* = 38 (IG^a^: 19; CG^b^: 19).  7–17 years. | 4 months. | Quasi-experimental study | Oculus Rift DK2 HMD (MOT^u^). | **Pain:**  VAS^f^. | + |
|  |  |  |  |  |  | **Stress:**  N/A^c^ | + |
| Aydın and Özyazıcıoğlu, 2019 [32]  **Turkey** | Venipuncture.  Hospital. | *n* = 120 (IG^a^: 60; CG^b^: 60).  9–12 years. | 5 months. | RTC^d^ | NAV^g^. | **Pain:**  VAS^f^  WBFPS^i^. | +  + |
| Caruso et al, 2019 [33]  **USA** | Venipuncture.  Hospital. | *n*=220 (IG^a^: 106; CG^b^: 114).  7–18 years. | 20 months. | RTC^d^ | Samsung Gear VR^e^ headset, (Ocean Rift, Pebbles the Penguin, or Space Pups). | **Pain:**  Pain scores.  FPS-R^j^. | –  – |
|  |  |  |  |  |  | **Fear:**  CFS^q^.  mICC^r^. | ±  ± |
|  |  |  |  |  |  | **Satisfaction surveys:**  5-point Likert agreement scale. | + |
|  |  |  |  |  |  | Adverse events to VR^e^ (nausea, vomiting, motion sickness, dizziness, or seizure). | N/A^c^ |
| Chen et al, 2020 [40]  **Taiwan** | Venipuncture.  Hospital. | *n*=136 (IG^a^: 68; CG^b^: 68).  7–12 years. | 6 months. | RTC^d^ | Xiaozhai V4 head-mounted display (age-appropriate VR^e^ apps downloaded through the App store). | **Pain:**  WBFPS^i^. | + |
|  |  |  |  |  |  | **Fear:**  CFS^q^. | + |
| Diaz-Hennessey et al, 2019 [41]  **USA** | Venipuncture.  Hospital. | *n*=15 (IG^a^: 8; CG^b^: 7).  8–18 years. | 14 weeks. | Quasi-experimental study. | Google Daydream VR^e^, Google Pixel cell phone (the application choices were: Wonderglade, Ocean Rift, Karts Sprint, Ace Fishing, or The Turning Forest). | **Pain:**  FLACC^s^.  Self-reported NRS^t^ scale. | + (5 min post-puncture).  – |
| Dumoulin et al, 2019 [42]  **Canada** | Venipuncture, intramuscular injections.  Hospital. | *n*=59 (TVG: 24; IG^a^: 20; CG^b^: 15).  8–17 years.  Mean age=13.37 years (*SD*=2.94). | N/A^c^ | RTC^d^ | NAV^g^, eMagin z800 HMD (VR^e^ intervention consisted of an immersive game developed by the UQO Cyberpsychology Lab using Virtools4). | **Pain, children and parents:**  VAS^f^. | + Children.  – Parents. |
|  |  |  |  |  |  | **Anxiety:**  VAS^f^ | + Children.  – Parents. |
|  |  |  |  |  |  | **Satisfaction:**  (scale 0–10). | + |
| Özkan and Polat, 2019 [47]  **Turkey** | Venipuncture.  Hospital. | *n*=135 (IG^a^ – VR^e^: 46; IG^a^ -Kaleidoscope: 46; CG^b^: 43).  4–10 years. | 6 months. | RTC^d^ | Samsung Gear VR^e^ Innovator edition goggle set, fitted with a Samsung Galaxy Note 4 mobile phone (NAV^g^) Samsung Gear glasses. Samsung Gear. | **Fear/Anxiety:**  CFS^q^. | + |
|  |  |  |  |  |  | **Pain:**  VAS^f^.  WBFPS^i^. | +  + |
| Walther-Larsen et al, 2019 [37]  **Denmark** | Venipuncture.  Hospital. | *n*=59 (IG^a^: 28; CG^b^: 31).  7–16 years. | 3 months. | RTC^d^ | Samsung galaxy S6 Gear VR^e^ goggles (Seagull Splash). | **Pain:**  VAS^f^. | – |
|  |  |  |  |  |  | **Satisfaction:**  Likert scale. Researcher developed questionnaire. | + |
|  |  |  |  |  |  | **Adverse effects:**  Nausea,  dizziness,  or other discomfort. | –  –  _ |
| Gerçeker et al, 2020 [44]  **Turkey** | Venipuncture.  Hospital. | *n*=136 (IG^a^ – VR^e^ 1:45; IG^a^ – VR^e^ 2:45; CG^b^: 46).  5–12 years. | 3 months. | RTC^d^. | IG^a^ – VR^e^ 1: Samsung: Gear Oculus with a Samsung Galaxy S5 Note Phone (rollercoaster).  IG^a^ – VR^e^ 2: Samsung: Gear Oculus (Ocean Rift). | **Pain:**  WBFPS^i^. | + |
|  |  |  |  |  |  | **Fear:**  CFS^q^. | + |
|  |  |  |  |  |  | **Anxiety:**  The children’s anxiety meter. | + |
| Piskorz et al, 2020 [34]  **Poland** | Venipuncture.  Hospital. | *n*=57 (Active- VR^e^; 19; Passive- VR^e^: 17; CG^b^: 21).  7-17 years. | 6 months | RTC^d^. | Active-VR^e^: Head-mounted display (Samsung gear), control de game (MOT^u^).  Passive-VR^e^: Head-mounted display (Samsung gear), observe the image (MOT^u^). | **Pain:**  VAS^f^  Attitude Towards blood sampling. Researchers developed. | +  - |
| Wong et al, 2020 [38]  **China** | Venipuncture.  Hospital. | *n*=108 (IG^a^: 54; CG^b^: 54).  6–17 years. | N/A^c^ | RTC^d^. | Google Cardboard Goggles (VR^e^ cartoons). | **Stress:**  VAS^f^ | + |
|  |  |  |  |  |  | **Anxiety:**  The State Anxiety Scale for Children (short version). | + |
|  |  |  |  |  |  | **Pulse Rate:**  Pulse oximeter. | – |
|  |  |  |  |  |  | **Procedure length:**  Standard stop watch. | + |
| Semerci et al, 2021 [38]  **Turkey** | Subcutaneous reservoir puncture.  Hospital. | *n*=71 (IG^a^: 35; CG^b^: 36).  7–18 years. | 11 months | RTC^d^. | The Piranha™ VR^e^ system (rollercoaster). VR headset.  NAV^g^ | **gPain:**  WBFPS^i^. | + |
| Goldman and Behboudi, 2021 [46]  **Canada** | Venipuncture.  Hospital. | *n*=66 (IG^a^: 35; CG^b^: 31).  6–16 years. | 4 months | RTC^d^. | VOX Z3 3D Virtual Reality Headset. Asus Zenfone 2 ZE551M (VR^e^ Roller Coaster app). | **Pain:**  FPS-R^j^. | +  – Change pre vs post |
|  |  |  |  |  |  | **Anxiety:**  Venham Situational Anxiety Score. | - |
|  |  |  |  |  |  | **Satisfaction:**  Open-ended questions. Researchers developed. | – Pain  + Anxiety |
| Erdogan and Ozdemir, 2021 [43]  **Canada** | Venipuncture.  Hospital. | *n*=142 (DC^w^; 35; VR^e^: 37; Buzzy®: 36; CG^b^: 34).  7–12 years. | 9 months | RTC^e^ | DC^w^: Distraction cards  VR^e^: (Samsung Galaxy Note 5 N920, Android 5.1.1, Lollipop Processor: Quad-core 1.5 GHz Cortex-A53 & Quad-core 2.1 GHz Cortex-A57), VR^e^ glasses (7.66 × 5.50 × 4.32 in., weigh 0.414 kg, Cyber, VR^e^ BOX 3.0), and a headset (Samsung Galaxy, microphone, Bluetooth, wired)    G2: Buzzy® (MMJ Labs, Atlanta, GA). | **Pain:**  WBFPS^i^.  VAS^f^ | + Cildren  + Children  + Parents |
|  |  |  |  |  |  | **Fear:**  CFS^q^. | + Children  + Parents |

*^a^IG, intervention group; ^b^CG, control group; ^c^N/A, not applicable; ^d^RTC, randomized trial controlled; ^e^VR, virtual reality; ^f^VAS, Visual Analogue Scale; ^g^ NAV, not available; ^h^CHEOPS, Children's Hospital of Eastern Ontario Pain Scale; ^i^ WBFPS, Wong-Baker faces pain rating scale; ^j^FPS-R, Faces Pain Scale–Revised; ^k^CAS, Color Analog Scale; ^l^OSBD, Observational Scale of Behavioral Distres; ^m^IPQ, I.V. Puncture Questionnaire; ^o^FAS, Fear, Anxiety, and Stress Scale; ^p^CASI, Child & Adolescent Symptom Inventory; ^q^CFS, Children’s Fear Scale; ^r^mICC, modified Induction Compliance Checklist; ^s^FLACC, Face, Legs, Activity, Cry, and Consolability; ^t^NRS, Numeric Rating Scale; ^u^MOT, multiple object tracking; ^v^SVE, simple virtual environment; ^w^DC, distraction card.*
