# Supplementary material for: Effect of Virtual Reality on Pediatric Pain and Fear During Procedures Involving Needles: Systematic Review and Meta-analysis
Source: JMIR Serious Games. 2022 Aug 9;10(3):e35008. doi: 10.2196/35008 (PMC9399850; doi:10.2196/35008)
Supplement: Multimedia Appendix 3 [file games_v10i3e35008_app3.doc]

**Multimedia Appendix 3. Evaluation of the methodological quality.**

|  |  | CASPE | | | | | | | | | | | JADAD |
| --- | --- | --- | --- | --- | --- | --- | --- | --- | --- | --- | --- | --- | --- |
|  |  |  | | | | | | | | | | |  |
| *Study* |  | *1. Did the study address a clearly focussed issue?* | *2. Was the assignment of participants random?* | *3. Were they adequately considered until the end of the study all participants?* | *4. Was the blinding maintained?* | *5.Were the groups similar at the start of the trial?* | *6. Were the groups treated equally?* | *7. How large was the treatment effect?^a^* | *8. What is the accuracy of this effect?^b^* | *9. Can these results be applied in our environment or local population?* | *10. Were all the results taken into account?* | *11. Do the benefits to be obtained justify the risks and costs?* |  |
| Wint et al, 2002 [50] |  | Yes | Yes (although it does not provide information on the randomization procedure). | Yes | No | Unclear | Yes | Low | Not reported | No (small sample size) | Yes | Yes | -1  Low |
| Gershon et al,  2004 [48] |  | Yes | Yes | Yes | No | Yes | Yes | Yes (only for anxiety; not to pain) | Not reported | No (small simple size with cancer) | Acceptability and satisfaction not considered | Yes | 1  Low |
| Wolitzky et al,  2005 [52] |  | Yes | Yes (although it does not provide information on the randomization procedure). | Yes | Unclear | Yes | Yes | Large | Not reported | No (small simple size) | Yes | Yes | -1  Low |
| Gold et al, 2006 [49] |  | Yes | Yes (although it does not provide information on the randomization procedure). | Yes | No | Yes | Yes | Low | Not reported | No (small sample size) | Yes | Yes | 1  Low |
| Windich-Biermeier et al,  2007 [51] |  | Yes | Yes | Yes | No | Yes | Yes | No | Not reported | No (small sample size and cancer children) | Yes | Yes | 1  Low |
| Gerçeker et al,  2018  [39] |  | Yes | Yes | Yes | No (only the child, parent, and nurse were blinded to each other's score) | Yes | Yes | Large | Not reported | Yes | Acceptability and satisfaction not considered | Yes | 2  Low |
| Gold and Mahrer 2018  [45] |  | Yes | Yes | Yes | Evaluators only. | Yes | Yes | Large | Not reported | Yes | Unclear | Yes | 4  High |
| Piskorz and Czub 2018 [35] |  | Yes | No | Yes | No | Unclear | Yes | Large | Not reported | No (small sample size) | Acceptability and satisfaction not considered | Yes | -1  Low |
| Aydin and Özyazıcıoglu 2019 [32] |  | Yes | Yes  (although it does not provide information on the randomization procedure). | Yes | Unclear | Yes | Yes | Large | Not reported | Yes | Acceptability and satisfaction not considered | Yes | 2  Low |
| Caruso et al, 2019 [33] |  | Yes | Yes | Yes | No | Yes | Yes | Low | Yes | Yes | Yes | Yes | 2  Low |
| Díaz-Hennessey et al,  2019 [41] |  | Yes | Uncertain | Yes | No | Unclear (no pre statistics reported) | Yes | Moderate to pain reported to evaluators  Not to pain reported to patients | Not reported | No (small simple size) | Acceptability and satisfaction not considered | Yes | -2  Low |
| Dumoulin et al,  2019 [42] |  | Yes | Yes | Yes | No | Yes | Yes | Insufficient results presented to determine | Not reported | No (small simple size) | Yes | Yes | -1  Low |
| Ozkan and Polat  2019 [47] |  | Yes | Yes | Yes | No | Yes | Yes | Large | Not reported | No | Acceptability and satisfaction not considered | Yes | 2  Low |
| Walther-Larsen et al, 2019 [37] |  | Yes | Yes | Yes | Blinded only to observers. | Yes | Yes | No | Not reported | Yes | Yes | Yes | 4  High |
| Chen et al, 2020 [40] |  | Yes | Yes | Yes | Unclear | No | Yes | Large | Not reported | Yes | Acceptability and satisfaction not considered | Yes | 2  Low |
| Gerçeker et al,  2020 [44] |  | Yes | Yes | Yes | Unclear | Yes | Yes | Large | Not reported | Yes | Acceptability and satisfaction not considered | Yes | 4  High |
| Piskorz et al, 2020 [34] |  | Yes | No | Yes | No | Unclear | No | Low | Not reported | No  (children with kidney failure) | Acceptability and satisfaction not considered | Yes | -2  Low |
| Wong et al, 2020 [38] |  | Yes | Yes | Yes | No | Yes | Yes | Large | Not reported | No (patients with cancer) | Yes | Yes | 2  Low |
| Semerci et al,  2021 [36] |  | Yes | Yes (by gender) | Yes | No | Yes | Yes | Large | Not reported | No (patients with cancer) | Acceptability and satisfaction not considered | Yes | 2  Low |
| Goldman and Behboudi 2021 [46] |  | Yes | Yes | Yes | NA | Unclear | Yes | Large | Not reported | No  (children hospitalized with different disease)) | Yes | Yes | 2  Low |
| Erdogan and Ozdemir  2021 [43] |  | Yes | Yes | Yes | No | Yes | Yes | Low | Reported | Yes | Acceptability and satisfaction not considered | Yes | 0  Low |

NA not applicable

aBased only on effect sizes for primary outcome (pain or anxiety) measures

bBased on confidence intervals around an effect size
